# Supplementary material for: Saprotrophic Wood Decay Ability and Plant Cell Wall Degrading Enzyme System of the White Rot Fungus Crucibulum laeve: Secretome, Metabolome and Genome Investigations
Source: J Fungi (Basel). 2024 Dec 31;11(1):21. doi: 10.3390/jof11010021 (PMC11766592; doi:10.3390/jof11010021)

Supplementary Figure S1. Exoproteome of *Crucibulum laeve* LE-BIN 1700 cultivated on: control glucose-peptone medium (**GP**); GP medium containing sawdust of alder (**GP-A**); GP medium containing sawdust of birch (**GP-B**); GP medium containing sawdust of pine (**GP-P**). For the data on MALDI TOF/TOF MS/MS analysis of the highlighted protein spots, please, refer to Figure 5.

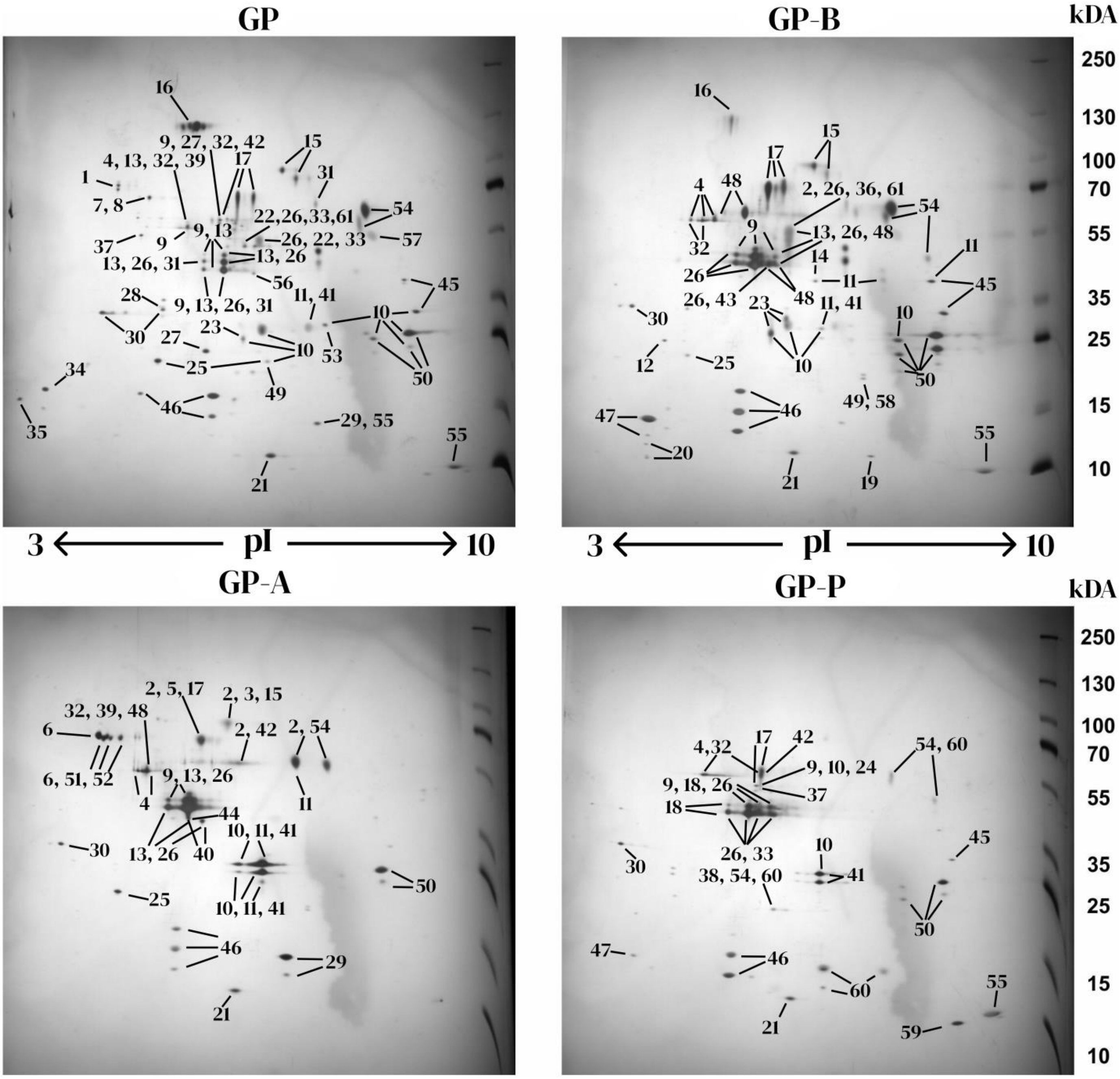

Supplement: Supplementary file 1 [file jof-11-00021-s001.zip › Supplementary Figure S1.pdf]
