# Supplementary material for: Saprotrophic Wood Decay Ability and Plant Cell Wall Degrading Enzyme System of the White Rot Fungus Crucibulum laeve: Secretome, Metabolome and Genome Investigations
Source: J Fungi (Basel). 2024 Dec 31;11(1):21. doi: 10.3390/jof11010021 (PMC11766592; doi:10.3390/jof11010021)

Supplementary Figure S2. Amino acid sequence alignment of TFK41392.1 and TFK41393.1 *Crucibulum laeve* proteins with LPMO-like copper protein X325 family from *Laetisaria arvalis* (MK088083). Strictly conserved amino acids are shown in red blocks. Residues of the semi-conservative GGDGN loop are framed in black frame; the strictly conserved residues – two histidines and Asp, involved in copper ion coordination in an active center of the enzyme are marked by asterisks; and the strictly four conserved cysteines forming two disulfide bonds are marked by triangles. The Asn residue involved in N-glycosylation is marked by black point.

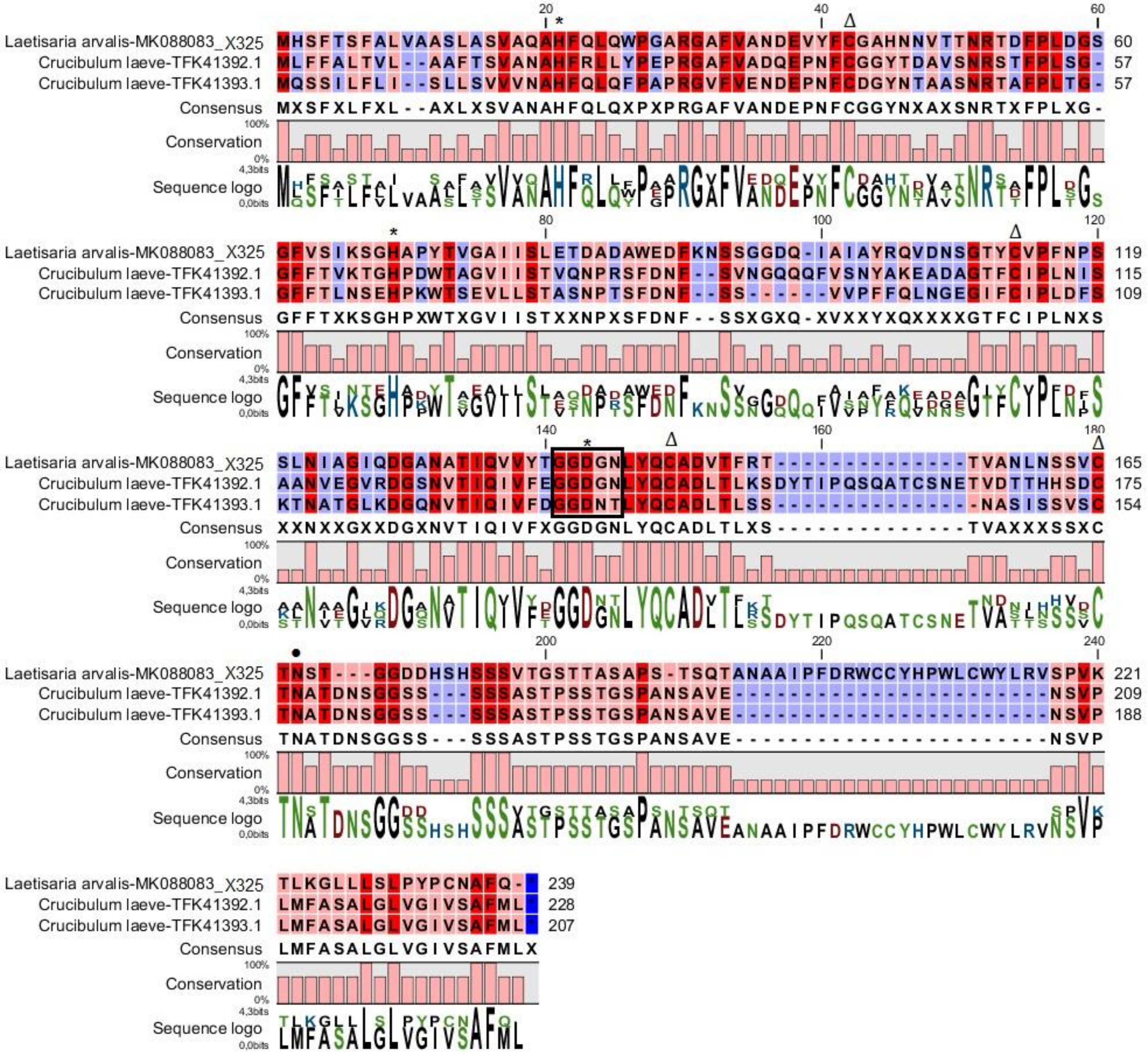

Supplement: Supplementary file 1 [file jof-11-00021-s001.zip › Supplementary Figure S2.pdf]
