# Supplementary material for: Saprotrophic Wood Decay Ability and Plant Cell Wall Degrading Enzyme System of the White Rot Fungus Crucibulum laeve: Secretome, Metabolome and Genome Investigations
Source: J Fungi (Basel). 2024 Dec 31;11(1):21. doi: 10.3390/jof11010021 (PMC11766592; doi:10.3390/jof11010021)

Supplementary Figure S3. Amino acid sequence alignment of TFK42532.1 and TFK42953.1 *Crucibulum laeve* proteins with fungal loosenins. Alignment includes *Bjerkandera adusta* Loos1 (GenBank code ADI72050.2), *Neurospora crassa* N2 (GenBank code XP_959591.1), *Phanerochaete carnosa* PcaLOOL2 (GenBank code EKM55357.1), *P. carnosa* LOOL7 (GenBank code EKM53490.1), *P. carnosa* LOOL9 (GenBank code EKM52742.1) and *P.carnosa* LOOL12 (GenBank code EKM51974.1). Strictly conserved amino acids are shown in red blocks and also include: 4 cysteines involved in formation of two disulfide bonds are marked by asterisks; the amino acids that form the shallow groove which potentially serves as a polysaccharide-binding site in D1 domen of expansins are marked by black points.

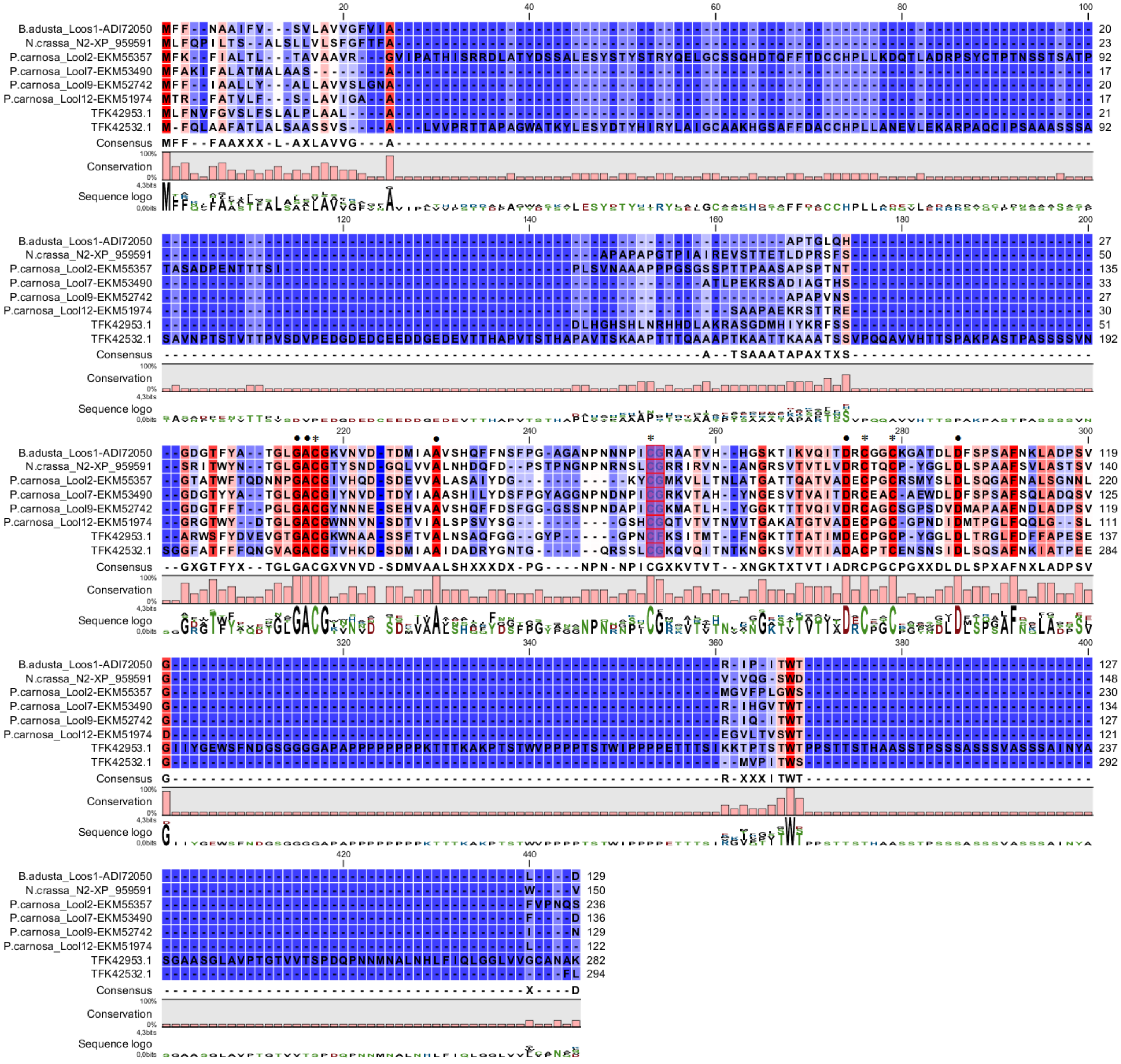

Supplement: Supplementary file 1 [file jof-11-00021-s001.zip › Supplementary Figure S3.pdf]
