# Supplementary material for: Saprotrophic Wood Decay Ability and Plant Cell Wall Degrading Enzyme System of the White Rot Fungus Crucibulum laeve: Secretome, Metabolome and Genome Investigations
Source: J Fungi (Basel). 2024 Dec 31;11(1):21. doi: 10.3390/jof11010021 (PMC11766592; doi:10.3390/jof11010021)

Supplementary Figure S4. GC-MS total ion current (TIC) chromatogram acquired from *Crucibulum leave* LE-BIN 1700 glucose-peptone (GP) culture liquid after derivatization with BSTFA. The list of metabolites corresponding to the numbered peaks on the GC/MS chromatogram, please, refer to Supplementary Table S1.

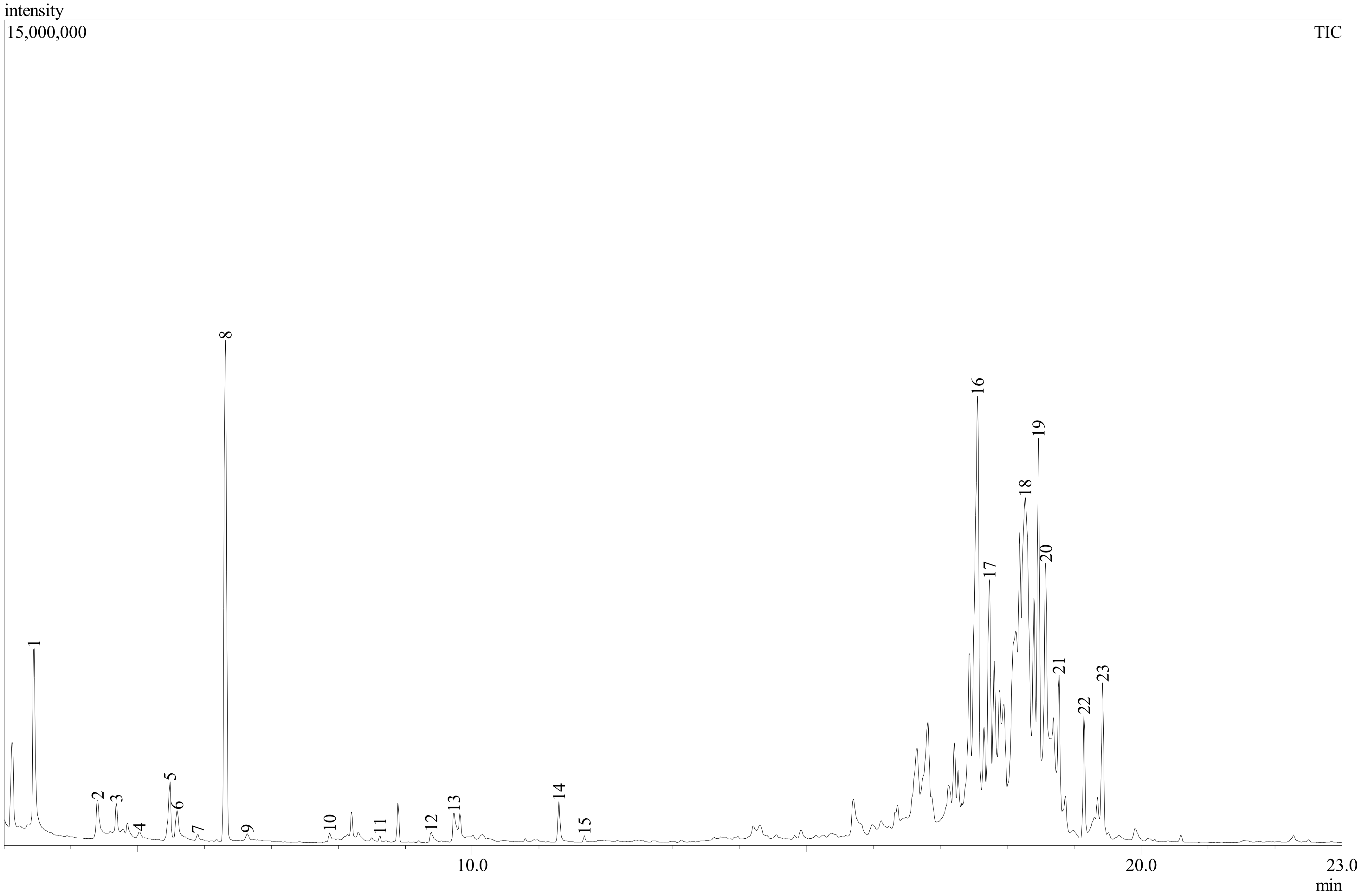

Supplementary Figure S5. GC-MS total ion current (TIC) chromatogram acquired from *Crucibulum leave* LE-BIN 1700 glucose-peptone with birch sawdust (GP-B) culture liquid after derivatization with BSTFA. The list of metabolites corresponding to the numbered peaks on the GC/MS chromatogram, please, refer to Supplementary Table S2.

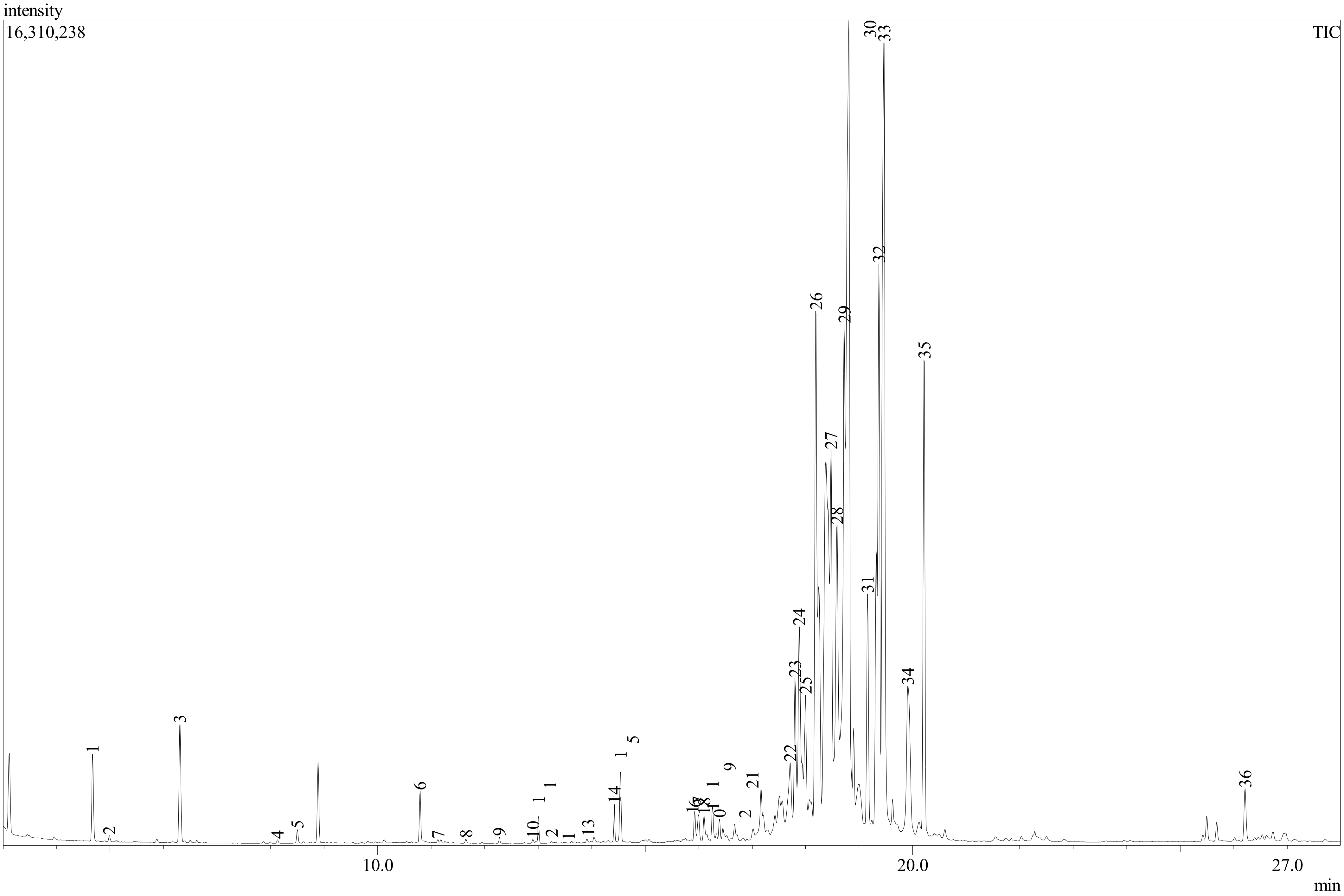

Supplementary Figure S6. GC-MS total ion current (TIC) chromatogram acquired from *Crucibulum leave* LE-BIN 1700 glucose-peptone with alder sawdust (GP-A) culture liquid after derivatization with BSTFA. The list of metabolites corresponding to the numbered peaks on the GC/MS chromatogram, please, refer to Supplementary Table S3.

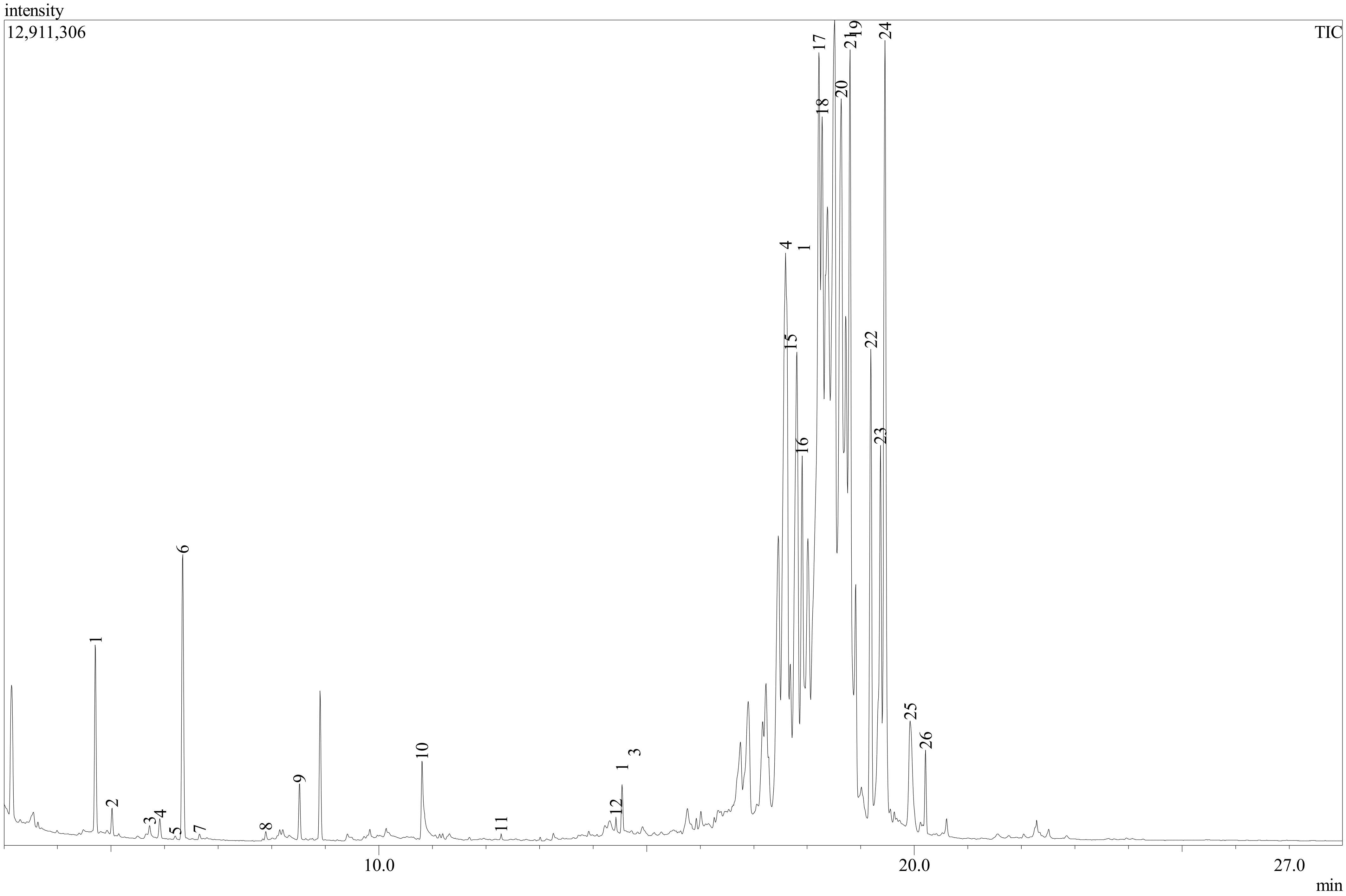

Supplementary Figure S7. GC-MS total ion current (TIC) chromatogram acquired from *Crucibulum leave* LE-BIN 1700 glucose-peptone with pine sawdust (GP-P) culture liquid after derivatization with BSTFA. The list of metabolites corresponding to the numbered peaks on the GC/MS chromatogram, please, refer to Supplementary Table S4.

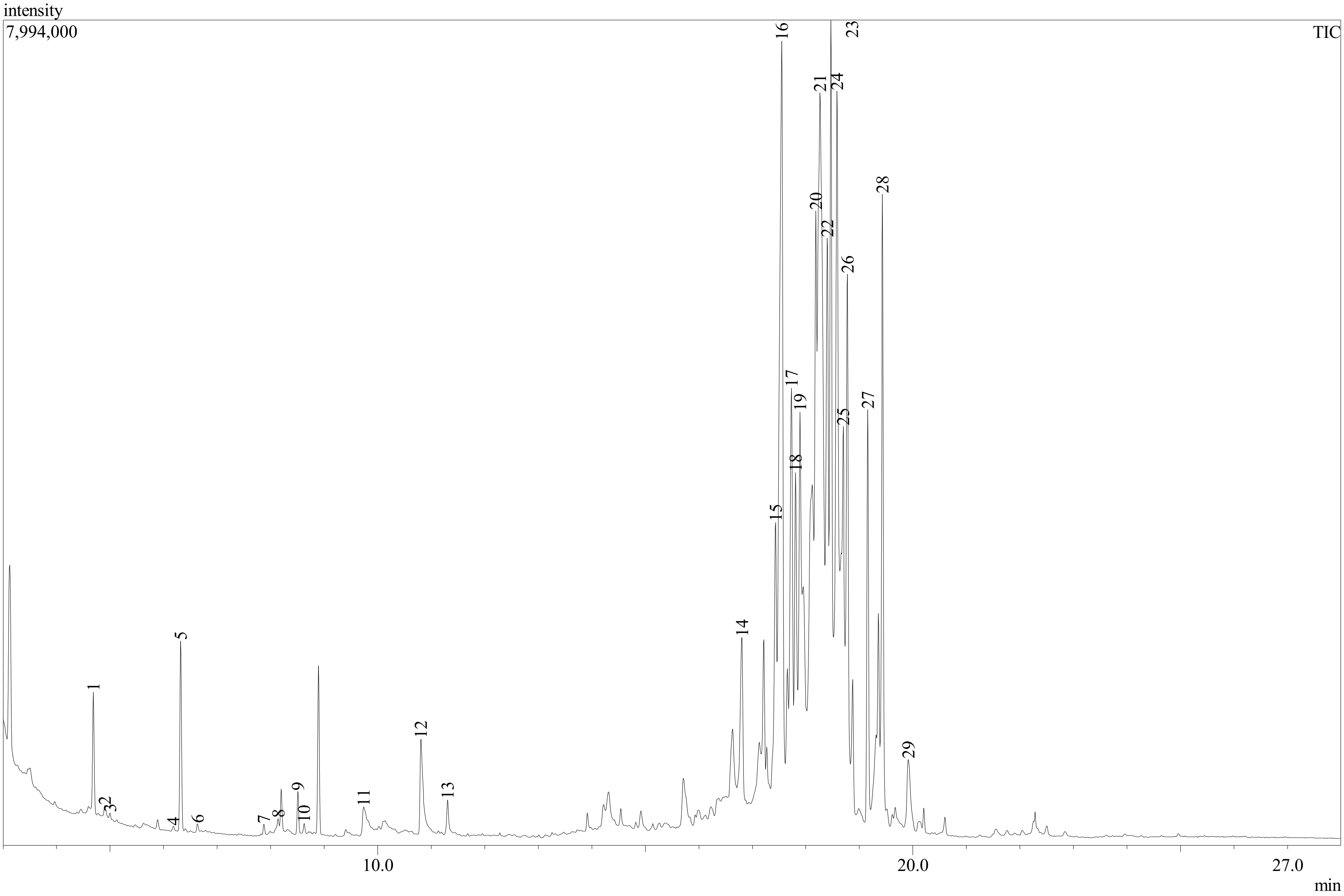

Supplement: Supplementary file 1 [file jof-11-00021-s001.zip › Supplementary Figure_S4-S7.pdf]
