# Supplementary material for: Saprotrophic Wood Decay Ability and Plant Cell Wall Degrading Enzyme System of the White Rot Fungus Crucibulum laeve: Secretome, Metabolome and Genome Investigations
Source: J Fungi (Basel). 2024 Dec 31;11(1):21. doi: 10.3390/jof11010021 (PMC11766592; doi:10.3390/jof11010021)
Supplement: Supplementary file 1 [file jof-11-00021-s001.zip › Supplementary Table S1-S4.pdf]

**Supplementary Table S1.** The results of the metabolomic analysis of the culture liquid of *Crucibulum leave* LE-BIN 1700 grown in the glucose-peptone medium (**GP**).

| **Peak #** | **RT** | **Area, %** | **Name** |
|---|---|---|---|
| 1 | 3.446 | 6.78 | D-(-)-Lactic acid, trimethylsilyl ester |
| 2 | 4.397 | 1.61 | 2-Butanol, trimethylsilyl ether |
| 3 | 4.679 | 0.83 | Silanol, trimethyl-, carbonate |
| 4 | 5.024 | 0.26 | Trimethyl(1-propoxypropan-2-yloxy)silane |
| 5 | 5.480 | 2.23 | D-(-)-Lactic acid, trimethylsilyl ether |
| 6 | 5.587 | 1.23 | Silane, (2-ethoxyethoxy)trimethyl- |
| 7 | 5.896 | 0.20 | Benzeneacetaldehyde |
| 8 | 6.309 | 19.34 | Propanoic acid, 2-[(trimethylsilyl)oxy]-, trimethylsilyl ester |
| 9 | 6.637 | 0.27 | Diethylene glycol, bistrimethylsilyl ether |
| 10 | 7.869 | 0.20 | 2-Propanone, bis(trimethylsilyloxy)- |
| 11 | 8.617 | 0.15 | Alpha-hydroxyisovaleric acid, (2TMS)- |
| 12 | 9.387 | 0.46 | 2-[(Trimethylsilyl)oxy]propan-1-ol |
| 13 | 9.726 | 1.56 | Hydroxy methyl furfural |
| 14 | 11.296 | 1.46 | Furan-2-carboxylic acid, 3-methyl-, trimethylsilyl ester |
| 15 | 11.677 | 0.19 | Diethylene glycol, bistrimethylsilyl ether |
| 16 | 17.555 | 20.20 | Hexadecanoic acid, trimethylsilyl ester |
| 17 | 17.733 | 7.12 | Glucofuranoside, methyl 2,3,5,6-tetrakis-O-(trimethylsilyl)-alpha-D- |
| 18 | 18.266 | 12.12 | Alpha-DL-Arabinofuranoside, methyl 2,3,5-tris-O-(trimethylsilyl)- |
| 19 | 18.466 | 7.53 | Beta-D-Galactopyranoside, methyl 2,4,6-tris-O-(trimethylsilyl)-, acetate |
| 20 | 18.572 | 6.06 | Glucofuranoside, methyl 2,3,5,6-tetrakis-O-(trimethylsilyl)-alpha-D- |
| 21 | 18.772 | 2.75 | Beta-L-Mannofuranose, 6-deoxy-1,2,3,5-tetrakis-O-(trimethylsilyl)- |
| 22 | 19.148 | 3.33 | Beta-D-Mannopyranoside, methyl 2,3,4,6-tetrakis-O-(trimethylsilyl)- |
| 23 | 19.425 | 4.11 | D-Galactose, 2,3,4,5,6-pentakis-O-(trimethylsilyl)- |

**Supplementary Table S2.** The results of the metabolomic analysis of the culture liquid of *Crucibulum leave* LE-BIN 1700 grown in the GP medium supplemented with birch sawdust (**GP-B**).

| Peak # | RT | Area, % | Name |
|---|---|---|---|
| 1 | 4.674 | 1.23 | Silanol, trimethyl-, carbonate |
| 2 | 4.985 | 0.07 | 3,6-Dioxa-2,7-disilaoctane, 2,2,4,7,7-pentamethyl- |
| 3 | 6.304 | 1.93 | Lactic acid, bis(trimethylsilyl)oxy-, ester |
| 4 | 8.131 | 0.05 | beta-Lactate di-TMS |
| 5 | 8.498 | 0.18 | Isobutyric acid, 3-trimethylsilyloxy-trimethylsilyl ester |
| 6 | 10.790 | 0.64 | Trimethylsilyl ether of glycerol |
| 7 | 11.127 | 0.04 | Butane, 1,2,3-tris(trimethylsiloxy)- |
| 8 | 11.653 | 0.05 | 3,7,11-Trioxa-2,12-disilatridecane, 2,2,12,12-tetramethyl- |
| 9 | 12.277 | 0.08 | Butane, 1,2,4-tris(trimethylsiloxy)- |
| 10 | 12.899 | 0.04 | D-(-)- Erythrofuranose, tris(trimethylsilyl) ether |
| 11 | 13.007 | 0.30 | D-(-)- Erythrofuranose, tris(trimethylsilyl) ether |
| 12 | 13.250 | 0.02 | 4-Trimethylsilyloxy-4-phenylbut-1-ene |
| 13 | 14.043 | 0.07 | Trimethylsilyl ether of glycerol |
| 14 | 14.423 | 0.44 | L-Threitol, tetrakis(trimethylsilyl) ether |
| 15 | 14.539 | 0.98 | Butane, 1,2,3,4-tetrakis[(trimethylsilyl)oxy]- |
| 16 | 15.928 | 0.35 | D-(-)-Ribofuranose, tetrakis(trimethylsilyl) ether (isomer 1) |
| 17 | 15.998 | 0.36 | Beta-DL-Arabinopyranose, 1,2,3,4-tetrakis-O-(trimethylsilyl)- |
| 18 | 16.101 | 0.29 | D-(-)-Lyxofuranose, tetrakis(trimethylsilyl) ether |
| 19 | 16.259 | 0.47 | D-(+)-Talofuranose, pentakis(trimethylsilyl) ether (isomer 1) |
| 20 | 16.387 | 0.24 | Beta-DL-Arabinopyranose, 1,2,3,4-tetrakis-O-(trimethylsilyl)- |
| 21 | 17.163 | 0.42 | Beta-D-Xylopyranose, 1,2,3,4-tetrakis-O-(trimethylsilyl)- |
| 22 | 17.709 | 1.40 | Alpha-DL-Arabinofuranoside, methyl 2,3,5-tris-O-(trimethylsilyl)- |
| 23 | 17.799 | 1.83 | Glucofuranoside, methyl 2,3,5,6-tetrakis-O-(trimethylsilyl)-alpha-D- |
| 24 | 17.877 | 2.45 | 1,5-Anhydro-D-sorbitol, tetrakis(trimethylsilyl) ether |
| 25 | 17.998 | 1.67 | D-(+)-Talofuranose, pentakis(trimethylsilyl) ether |
| 26 | 18.191 | 6.23 | Glucofuranoside, methyl 2,3,5,6-tetrakis-O-(trimethylsilyl)-alpha-D- |
| 27 | 18.472 | 5.09 | Glucofuranoside, methyl 2,3,5,6-tetrakis-O-(trimethylsilyl)-alpha-D- |
| 28 | 18.582 | 4.22 | Alpha-DL-Arabinofuranoside, methyl 2,3,5-tris-O-(trimethylsilyl)- |
| 29 | 18.722 | 8.02 | L-(+)-Rhamnopyranose, tetrakis(trimethylsilyl) ether |
| 30 | 18.807 | 18.95 | Beta-L-Mannofuranose, 6-deoxy-1,2,3,5-tetrakis-O-(trimethylsilyl)- |
| 31 | 19.156 | 3.60 | Beta-D-Mannopyranoside, methyl 2,3,4,6-tetrakis-O-(trimethylsilyl)- |
| 32 | 19.365 | 7.92 | 2-alpha-Mannobiose, octakis(trimethylsilyl) ether (isomer 1) |
| 33 | 19.466 | 17.48 | D-Galactose, 2,3,4,5,6-pentakis-O-(trimethylsilyl) |
| 34 | 19.909 | 4.28 | Beta.-DL-Arabinopyranose, 1,2,3,4-tetrakis-O-(trimethylsilyl)- |
| 35 | 20.216 | 7.70 | Beta-D-Glucopyranose, 1,2,3,4,6-pentakis-O-(trimethylsilyl)- |
| 36 | 26.211 | 0.91 | 3-.alpha.-Mannobiose, octakis(trimethylsilyl) ether (isomer 2) |

**Supplementary Table S3.** The results of the metabolomic analysis of the culture liquid of *Crucibulum leave* LE-BIN 1700 grown in the GP medium supplemented with alder sawdust (**GP-A**).

| Peak # | RT | Area, % | Name |
|---|---|---|---|
| 1 | 4.705 | 2.46 | Silanol, trimethyl-, carbonate |
| 2 | 5.014 | 0.32 | 3,6-Dioxa-2,7-disilaoctane, 2,2,4,7,7-pentamethyl- |
| 3 | 5.716 | 0.11 | Butane, 2,3-bis(trimethylsiloxy)- |
| 4 | 5.911 | 0.27 | Butane, 2,3-bis(trimethylsiloxy)- |
| 5 | 6.196 | 0.04 | 1,3 Propanediol di-TMS |
| 6 | 6.334 | 4.35 | Lactic acid, bis(trimethylsilyl)oxy-, ester |
| 7 | 6.653 | 0.07 | Glycolic acid, bis-TMS |
| 8 | 7.885 | 0.08 | 3,7-Dioxa-2,8-disilanonan-5-one, 2,2,8,8-tetramethyl- |
| 9 | 8.516 | 0.71 | Isobutyric acid, 3-trimethylsilyloxy-trimethylsilyl ester |
| 10 | 10.803 | 1.68 | Trimethylsilyl ether of glycerol |
| 11 | 12.285 | 0.06 | Butane, 1,2,4-tris(trimethylsiloxy)- |
| 12 | 14.428 | 0.16 | meso-Erythritol, tetrakis(trimethylsilyl) ether |
| 13 | 14.544 | 0.61 | L-Threitol, tetrakis(trimethylsilyl) ether |
| 14 | 17.595 | 14.67 | Octanoic acid, trimethylsilyl ester |
| 15 | 17.802 | 9.51 | Beta-D-Galactopyranoside, methyl 2,4-bis-O-(trimethylsilyl)-diacetate |
| 16 | 17.901 | 3.60 | Glucofuranoside, methyl 2,3,5,6-tetrakis-O-(trimethylsilyl)-alpha-D- |
| 17 | 18.220 | 4.77 | Beta-D-Galactopyranoside, methyl 2,4,6-tris-O-(trimethylsilyl)-acetate |
| 18 | 18.278 | 2.35 | Glucofuranoside, methyl 2,3,5,6-tetrakis-O-(trimethylsilyl)-alpha-D- |
| 19 | 18.508 | 10.89 | Beta-D-Galactopyranoside, methyl 2,4,6-tris-O-(trimethylsilyl)-acetate |
| 20 | 18.630 | 8.53 | D-(-)-Ribofuranose, tetrakis(trimethylsilyl) ether (isomer 1) |
| 21 | 18.800 | 6.90 | Glucofuranoside, methyl 2,3,5,6-tetrakis-O-(trimethylsilyl)-alpha-D- |
| 22 | 19.186 | 7.55 | Beta-D-Mannopyranoside, methyl 2,3,4,6-tetrakis-O-(trimethylsilyl)- |
| 23 | 19.364 | 3.19 | Glucofuranoside, methyl 2,3,5,6-tetrakis-O-(trimethylsilyl)-alpha-D- |
| 24 | 19.454 | 13.03 | D-Galactose, 2,3,4,5,6-pentakis-O-(trimethylsilyl)- |
| 25 | 19.919 | 3.20 | D-(-)-Ribofuranose, tetrakis(trimethylsilyl) ether |
| 26 | 20.211 | 0.88 | Beta-D-Glucopyranose, 1,2,3,4,6-pentakis-O-(trimethylsilyl)- |

**Supplementary Table S4**. The results of the metabolomic analysis of the culture liquid of *Crucibulum leave* LE-BIN 1700 grown in the GP medium supplemented with pine sawdust (**GP-P**).

| **Peak #** | **RT** | **Area, %** | **Name** |
|---|---|---|---|
| 1 | 4.687 | 1.31 | Silanol, trimethyl-, carbonate |
| 2 | 4.900 | 0.14 | Silane, (2-furanylmethoxy)trimethyl- |
| 3 | 4.994 | 0.05 | 3,6-Dioxa-2,7-disilaoctane, 2,2,4,7,7-pentamethyl- |
| 4 | 6.182 | 0.05 | 1,3 Propanediol di-TMS |
| 5 | 6.317 | 2.27 | Lactic acid, bis(trimethylsilyl)oxy-ester |
| 6 | 6.635 | 0.09 | Glycolic acid, bis-TMS |
| 7 | 7.871 | 0.11 | 3,7-Dioxa-2,8-disilanonan-5-one, 2,2,8,8-tetramethyl- |
| 8 | 8.140 | 0.07 | 2-Methyl-1,3-bis(trimethylsiloxy)propane |
| 9 | 8.507 | 0.46 | Isobutyric acid, 3-trimethylsilyloxy-trimethylsilyl ester |
| 10 | 8.623 | 0.11 | Pentanoic acid, 2-[(trimethylsilyl)oxy]-trimethylsilyl ester |
| 11 | 9.738 | 0.78 | 5-Hydroxymethylfurfural |
| 12 | 10.804 | 2.34 | Trimethylsilyl ether of glycerol |
| 13 | 11.304 | 0.51 | Furan-2-carboxylic acid, 3-methyl-trimethylsilyl ester |
| 14 | 16.804 | 2.83 | Butyric acid, 4-ethoxy-, trimethylsilyl ester |
| 15 | 17.434 | 2.17 | alpha-D-Glucopyranose, 2-amino-3,6-anhydro-2-deoxy-1,4-bis-O-(trimethylsilyl)- |
| 16 | 17.549 | 14.81 | Fatty acid |
| 17 | 17.728 | 4.71 | Beta-D-Galactopyranoside, methyl 2,4,6-tris-O-(trimethylsilyl)-acetate |
| 18 | 17.805 | 2.51 | Beta-D-Galactopyranoside, methyl 2,4,6-tris-O-(trimethylsilyl)-acetate |
| 19 | 17.887 | 2.60 | Glucofuranoside, methyl 2,3,5,6-tetrakis-O-(trimethylsilyl)-alpha-D- |
| 20 | 18.187 | 3.95 | Glucofuranoside, methyl 2,3,5,6-tetrakis-O-(trimethylsilyl)-alpha-D- |
| 21 | 18.265 | 13.98 | Glucofuranoside, methyl 2,3,5,6-tetrakis-O-(trimethylsilyl)-alpha-D- |
| 22 | 18.400 | 4.03 | Alpha-DL-Arabinofuranoside, methyl 2,3,5-tris-O-(trimethylsilyl)- |
| 23 | 18.467 | 7.71 | Beta-D-Galactopyranoside, methyl 2,4,6-tris-O-(trimethylsilyl)-, acetate |
| 24 | 18.579 | 8.53 | D-(-)-Ribofuranose, tetrakis(trimethylsilyl) ether (isomer 1) |
| 25 | 18.695 | 2.59 | Beta-D-Mannopyranoside, methyl 2,3,4,6-tetrakis-O-(trimethylsilyl)- |
| 26 | 18.777 | 6.76 | Glucofuranoside, methyl 2,3,5,6-tetrakis-O-(trimethylsilyl)-alpha-D- |
| 27 | 19.154 | 5.00 | Beta-D-Xylopyranose, 1,2,3,4-tetrakis-O-(trimethylsilyl)- |
| 28 | 19.432 | 7.93 | Galactose, penta-TMS-ether |
| 29 | 19.911 | 1.61 | D-(-)-Ribofuranose, tetrakis(trimethylsilyl) ether |
